# Production of transgenic first filial puppies expressing mutated human amyloid precursor protein gene

**DOI:** 10.3389/fvets.2023.1227202

**Published:** 2023-10-30

**Authors:** Mohammad Shamim Hossein, Young-Bum Son, Yeon Woo Jeong, Yeon Ik Jeong, Mi Na Kang, Eun Ji Choi, Kang Bae Park, Yu Ra Bae, Dae Young Kim, Woo Suk Hwang

**Affiliations:** ^1^UAE Biotech Research Centre, Abu Dhabi, United Arab Emirates; ^2^Department of Obstetrics, College of Veterinary Medicine, Chonnam National University, Gwangju, Republic of Korea; ^3^Department of Companion Animal and Animal Resources Science, Joongbu University, Geumsan-gun, Republic of Korea; ^4^Department of Life Science, College of Bio-nano Technology, Gachon University, Seongnam, Republic of Korea; ^5^Department of Biology, North-Eastern Federal University, Yakutsk, Russia

**Keywords:** transgenic dogs, mhAPP gene, germline transmission, Percoll, *in vitro* fertilization

## Abstract

Propagation of transgenic animals by germline transmission using assisted reproductive technologies such as *in vitro* fertilization (IVF) is the most efficient way to produce transgenic colonies for biomedical research. The objective of this study was to generate transgenic puppies from a founder dog expressing the mutated human amyloid precursor protein (mhAPP) gene. Experiment I assessed the characteristics of the semen prepared by freshly diluted, swim-up, and Percoll gradient methods using a computer-assisted semen analyzer (CASA). Motile and progressively motile sperm counts were higher in the Percoll gradient samples (*p* < 0.05) than in the swim-up and freshly diluted samples. In Experiment II, a total of 59, 70, and 65 presumptive zygotes produced by fresh, Percoll gradient, and swim-up methods, respectively, were transferred to surrogates (5 for each group); the Percoll gradient (27.27%) and swim-up samples (14.29%) showed the highest blastocyst formation rates, while fresh diluted semen did not produce any blastocyst. Experiment III examined the full-term developmental ability of embryos. Among the 5 surrogates in the Percoll gradient group, one (20.0%) became pregnant; it had 4 (6.15%) sacs and delivered 4 (6.15%; 2 males and 2 females) live puppies. Among the 4 puppies, 2 (50.0%) were found to transmit the transgene on their nail and toe under GFP fluorescence. Furthermore, the integration and expression of the mhAPP transgene were examined in the umbilical cords of all the IVF-derived puppies, and the presence of the transgene was only observed in the GFP-positive puppies. Thus, semen prepared by the Percoll method could generate transgenic puppies by male germline transmission using the IVF technique. Our result will help propagate transgenic dogs efficiently, which will foster human biomedical research.

## 1. Introduction

Transgenic animals are useful in studying human diseases as they can provide deep insights into the molecular mechanisms contributing to the pathogenesis of specific diseases. It could assist in developing improved treatment regimens by identifying agents that can abrogate the onset of the disease, slow its progression, or alleviate its symptoms. However, selecting appropriate animals is vital in disease model experiments as closer genetic and physiological similarities with humans could generate more valuable and usable information (1). Although mice are widely used in transgenic models and have contributed immensely to human biomedical science, the results obtained from the mouse model cannot directly apply to humans due to huge physiological and genetic differences (2). Canine models can overcome some of those issues as they have close physiological and genetic similarities with humans (3). Furthermore, dogs live with humans in the same environment and share similar food, which makes them unique candidates. Dogs exhibit more than 600 genetic defects, and of those, 400 have a high degree of similarity with humans (4, 5). In particular, transgenic dogs are a powerful model for preclinical assessment of the safety and efficacy of stem cell or gene therapy for human diseases.

The first transgenic animals were generated by pronuclear microinjection (6). After that, various methods such as sperm-mediated gene transfer (7), lentiviral transgene delivery (8), and CRISPR/Cas9 system (9) were used to produce transgenic animals. Somatic cell nuclear transfer (SCNT) is the most versatile tool for producing transgenic animals as it has greater efficiency (10). Using the SCNT, we generated transgenic dogs overexpressing mutated human amyloid precursor protein (mhAPP) (11). Dogs overexpressing the mhAPP gene may be used to understand the pathological and developmental characteristics of Alzheimer’s disease in humans (12). It is essential to propagate the founder animals to develop specialized transgenic colonies as many animals are needed for biomedical research to avoid statistical bias and to obtain more precise information. SCNT can produce many duplicates of genetically engineered animals bypassing conventional breeding procedures. However, some reproductive complexities, such as ovulating immature oocytes and lack of a reliable *in vitro* maturation system, make canine cloning more challenging than other species (13, 14). The time, cost, and procedural intricacy related to SCNT could be reduced significantly if transgenic puppies could be obtained from founder transgenic dogs using natural breeding or assisted reproductive technologies (ART).

The reproductive abilities of male and female transgenic dogs have been thoroughly investigated, and it is reported that they have standard reproductive capabilities (15). Hence, they can be propagated naturally by germline transmission, where transgene can be incorporated with sperm or oocytes and genetically transferred to their descendants. Germline transmission is the most efficient way to produce transgenic colonies in a shorter period at a relatively low cost (15). The first transgenic puppy produced by germline transmission by natural mating was reported by Hong et al. (15); they produced offspring using cloned transgenic female dogs. However, male transgenic animals are more suitable because when transgenic semen is combined with IVF technologies, it is possible to produce transgenic offspring more efficiently. To the best of our knowledge, no studies have described the successful germline transmission of transgenes to progeny using the IVF technique in canines.

IVF is a well-established reproductive technique that can be used in mammals for a variety of reasons. In humans, it is primarily used to overcome male or female infertility-related problems (16). In farm animals, an increase in selection intensity is the leading cause of IVF. Although puppies have been born from IVF before (17), the overall usage of IVF in canines is far lower than that of other mammals. Successful IVF requires an improved sperm preparation method as semen may have both functional and morphological abnormal spermatozoa. Sperm preparation methods that improve the selection of spermatozoa with normal morphology, intact acrosomes, high curvilinear or straight-line velocity, and linearity are positively correlated with successful IVF (18). Various sperm separation methods have been developed and successfully used for decades in different species. Among them, the swim-up and Percoll gradient methods are the most widely applied (16, 19). However, the results are not always consistent. Mammalian spermatozoa differ in size, shape, and weight and species-specific differences have been reported. A comparison of these common sperm preparation methods still needs to be performed for canines. This study aimed to produce transgenic puppies by germline transmission using IVF techniques and compare two semen preparation methods for canine IVF, the Percoll gradient, and swim-up methods using a computer-assisted semen analyzer (CASA, Hamilton Thorne v.12.4 Build 006A HTC).

## 2. Materials and methods

### 2.1. Care and management of animals

A total of 12 mixed-breed pro-estrus dogs aged between 1 and 7 years with a body weight of 20–25 kg were obtained from a breeder. Among them, seven dogs were used as oocyte donors, and five were recipients. During experiments, dogs were reared in indoor kennels (2.5 × 1.5 m). The pregnant recipients were kept in the research facility, and non-pregnant and oocyte donors were returned to the breeder. Commercial food was given to dogs once daily, and water was provided *ad libitum*.

Licensed veterinarians have performed all surgical procedures under general anesthesia. All surgical procedures were conducted following the animal study guidelines, which the ethics committee approved at the Abu Dhabi Biotech Research Foundation, Republic of Korea (Permit no. C-16-01). We used propofol (1 mg/kg body weight) to induce the anesthesia and 2% isoflurane inhalation to maintain the anesthesia.

### 2.2. Chemicals and reagents

Unless otherwise mentioned, we purchased all chemicals and reagents from Sigma-Aldrich (St. Louis, MO, United States).

### 2.3. Collection of *in vivo* matured oocyte

Ovulation time was determined by measuring the serum progesterone concentration once daily and by ovarian ultrasonography (20, 21). The *in vivo* matured oocytes were collected by flushing both fallopian tubes at 72–84 h after ovulation. The reproductive tracts were accessed by mid-ventral laparotomy. The flushing medium was prepared using TCM 199 supplemented with HEPES (4.7 mg/mL), 10% (v/v) fetal bovine serum (FBS, Thermo Fisher Scientific, Waltham, MA, United States), and 1% (v/v) antibiotic-antimycotic (Gibco, Thermo Fisher Scientific, Waltham, MA, United States).

### 2.4. Semen preparation

A 27-month-old transgenic dog expressing the mhAPP gene was used as the semen donor. The dog was clinically healthy, and the semen parameter was within the normal range. After 3 days of abstinence, the second sperm-rich fraction of ejaculate was collected by manual stimulation. Semen was washed by centrifugation at 750 × g for 5 min in wash-TALP (TALP: Tyrode’s albumin lactate pyruvate) and divided into three fractions. Semen was then prepared for IVF using three different methods: freshly diluted, Percoll gradient, and swim-up procedures for further experimentation. The composition of wash-TALP, Cap-TALP (capacitation-TALP), and IVF-TALP is given in Supplementary Table S1, which is a slight modification of Mahi and Yanagimachi (22) and Nagashima et al. (17).

#### 2.4.1. Fresh dilution

The semen was centrifuged at 400 × g for 5 min to pellet the sperm. Sperm were then resuspended in Cap-TALP and incubated at a concentration of 7.5×10^6^ sperm/mL for 4 h at 38°C with 5% CO_2_ for capacitation.

#### 2.4.2. Swim-up procedure

Semen was prepared for the swim-up procedure as previously described by Parrish et al. (23). Briefly, fresh semen (0.5 mL) was layered in duplicate (0.25 mL) under 1 mL of Cap-TALP and incubated for 30 min at 38°C in a humidified atmosphere of 5% CO_2_ and 95% air. The top 0.80 mL of medium from each tube was collected, pooled, and centrifuged at 1000 × g for 10 min. The pellet was washed by centrifugation in fresh Cap-TALP, the final pellet was reconstituted in 0.5 mL of Cap-TALP, and its concentration was adjusted to 7.5×10^6^ sperm/mL.

#### 2.4.3. Percoll gradient procedure

Semen was prepared for the swim-up procedure as previously described (23). A 90% (v/v) and 45% (v/v) isotonic Percoll solution was prepared by adding 5 mL of Cap-TALP (capacitation -TALP) to 45 mL of Percoll and 1 mL Cap-TALP to 1 mL of 90% Percoll, respectively. Percoll gradient was prepared in a 15 mL conical tube by adding 1 mL of 90% Percoll to the bottom of the tube and 2 mL of 45% Percoll on its top. Finally, 0.5 mL of semen was placed carefully on top of the Percoll gradient without mixing the layers. The tube was then centrifuged at 700 × g for 10 min. The pellet was diluted in 1 mL Cap-TALP, and the second washing was done by centrifugation at 700 × g for 5 min. The pellet was diluted by adjusting sperm concentration to 7.5×10^6^ sperm/mL using Cap-TALP.

### 2.5. Measurement of sperm characteristics

Experiment I was conducted to assess the various characteristics of spermatozoa associated with their fertility in each group. Nine parameters, namely, motility, progressive motility, average path velocity (VAP), linear velocity (VSL), curvilinear velocity (VCL), straightness coefficient (STR), linear coefficient (LIN), mean amplitude of head lateral displacement (mALH), and frequency of head displacement (BCF), were measured using CASA (Hamilton Thorne Inc., Beverly, United States). This device consists of a phase-contrast microscope, a camera, a minitherm stage warmer, and an image digitizer. The settings for the canine sperm samples were established using the method described in Rijsselaere et al. (24). Sperm samples (8 μL) were placed in a cell chamber (Leja Products B.V., depth 20 μm) under a microscope, and the tracks from at least 1,000 sperm were recorded and evaluated. On average, 4 images were evaluated in 32 frames per analysis at 20 ms intervals. The Cell Motion Analyzer 2.0 software (CMA, Medical Technologies; Montreux, Switzerland) was used to evaluate the percentage of motile sperm and track velocities.

### 2.6. *In vitro* fertilization and embryo transfer

Prior to IVF, oocytes were washed several times in IVF-TALP. A group of 6–8 oocytes with homogenously dark cytoplasm and normal morphology in 6 μL of IVF-TALP were transferred into a 40 μL of IVF-TALP droplet. The IVF droplets were overlaid with light mineral oil; 4 μL of sperm suspension (3.0×10^4^ sperm/ drop) was added to each droplet and co-incubated for 4 h at 5% CO2 and 38.5°C in humidified air. After 4 h, oocytes were pipetted gently in IVF-TALP medium to remove adherent sperm and transferred to pre-equilibrated IVF-TALP droplets. After co-incubation for 12 h, oocytes were denuded in TCM 199 supplemented with HEPES (4.7 mg/mL), 10% (v/v) FBS, and 1% (v/v) antibiotic-antimycotic.

The ovary was approached using a mid-ventral laparotomy. The fallopian tube corresponding to the ovary with more corpora lutea was selected for embryo transfer. Presumptive zygotes were loaded into a 3.5 Fr × 5.5” Sterile Tom Cat Catheter (Argyle™ Covidien, Dublin, Ireland) in 2–4 μL IVF-TALP and gently advanced 2.5 cm (2/3 distal position) into the oviduct via the infundibulum and zygotes were slowly dispensed.

### 2.7. Embryo collection

Experiment II was conducted to examine the *in vivo* pre-implantation development of the embryos. Embryos were collected on day 7 post-transfer (10 days after ovulation) as previously described (25). Briefly, a Foley balloon catheter (FBC) was used for flushing the reproductive tracts using a balloon tip catheter without excision. Uterine and oviduct flushings were performed under general anesthesia on day 7 post-embryo transfer (ET). After surgical exposure of the reproductive tract, a needle puncture was made in the uterine body using a fine needle, which was then removed and replaced with a balloon catheter caudal to the bifurcation of the uterus. After ligating the FBC into the cervix and inflating the balloon, the upper part of the designated segment of the uterus was ligated to prevent backflow and the needle was inserted allowing 10 mL TCM 199 supplemented with HEPES flushing medium to flow through three or four times. After collection, the perforation was closed using a single stitch.

### 2.8. Pregnancy diagnosis

In Experiment III, the full-term developmental ability of IVF-derived embryos was evaluated. Pregnancy was diagnosed using a portable ultrasound machine with a 3.5 MHz curved transducer (SonoAce R7; Samsung Medison, Seoul, Korea) after 25 days of ET in either the standing or dorsal recumbence position. Ultrasonography was repeated weekly to monitor fetal development.

### 2.9. Genomic DNA extraction, RT-qPCR

G-DEX™ II Genomic DNA Extraction kit (Intron Biotechnology, Suwon, South Korea) was used to isolate canine genomic DNA. After isolation, 100 ng of DNA was amplified using 10 pmol of specific primers in a 20 μL PCR mixture having 2× Lamp Taq PCR Master Mix 3 (BioFact, Daejeon, Korea). We used the PCR condition as follows: 35 denaturation cycles each for 30 s at 95°C, annealing for 30 s at 55°C after that, extension for 30 s at 72°C, and a final extension for 5 min at 72°C. We separated the PCR product on 1.5% agarose gel after staining with EcoDye™ Nucleic acid staining solution (BioFact, Daejeon, Korea). The photographs were taken using UV illumination, and images were scanned using a Gel Doc EQ system (Bio-Rad Laboratories, Inc., Hercules, CA, United States).

The relative copy number of the mhAPP gene in 4 transgenic puppies was compared to the original transgenic semen donor dog (SBM16). The experiment was repeated 3 times in a 20 μL reaction volume mixture consisting of 2× SYBR Premix Ex Taq (Takara Bio, Inc., Otsu, Japan) and 10 pmol of specific primers on a 7,500 real-time PCR System (Applied Biosystems, Foster City, CA, United States). We used 1 cycle of 10 min at 95°C, and 40 cycles of 15 s at 95°C, 15 s at 55°C, and 30 s at 72°C as amplification parameters for real-time quantitative PCR (RT-qPCR). At the end of the amplification, a melting curve analysis was recorded to confirm that there was no contamination by primer dimers. Standard curves were established using 5 serial dilutions of a reference plasmid. Fluorescence was acquired to determine the threshold cycle during the log-linear phase of the reaction at which fluorescence rose above the background. The DNA concentration was measured by using a 7,500 real-time PCR (qPCR). The qPCR uses real-time fluorescence to measure the quantity of DNA present at each cycle during PCR. Data obtained by qPCR were plotted in Excel, and the ΔΔCt method was used to calculate the relative concentration of gene expressed compared with GAPDH. Details of the primers used in this study are presented in Table 1.

**Table 1:** List of primers.

Gene	Primer	bp	Accession number/reference
GAPDH	Forward primer 5'-GGAGAAAGCTGCCAAATATGACG -3'	118	NM_001003142.2
	Reverse primer 5'-ACTGTTGAAGTCACAGGAGACC -3'		
mhAPP	Forward primer 5'-CCTTGTGCTGTCTCCCCCTC-3'	834	Lee et al. (11)
	Reverse primer 5'- TCACAAAGTGGGGATGGGTC -3'		

### 2.10. Parentage analysis

Genomic DNA was isolated from the blood of the sperm donor, the umbilical cords of offspring, and the skin of recipients and oocyte donors using a QIAamp DNA Micro Kit (Qiagen, Hilden, Germany) following the manufacturer’s instructions. Parentage was confirmed using short tandem repeat (STR) profiling using 18 canine-specific microsatellite loci (AHTk211, AHTh260, AHTk253, AHTh171, AHT137, AHT121, CXX279, FH2848, FH2054, INRA21, INU055, INU005, INU030, REN54P11, REN169018, REN169D01, REN162C04, and REN247M23). The amplified fragments were analyzed using ABI 3130xL Genetic Analyzer (Applied Biosystems, Foster City, CA, United States) using POP-4 Polymer (Applied Biosystems), with the base pair lengths were scored using GeneScan Software v 3.2 (Applied Biosystems).

### 2.11. Statistical analysis

We used the SPSS statistical program (SPSS Inc., Chicago, IL, United States) for statistical analyses. A one-way analysis of variance (ANOVA) followed by Duncan’s post-hoc test was used to analyze the sperm parameters, pre-implantation developmental competence of IVF-derived embryos, and relative mhAPP copy number. Blastocyst quality and pregnancy rate were compared using Pearson’s chi-square test and Fisher’s exact test. The normal distribution of data was evaluated before performing statistical analysis. GraphPad Prism (version 4.0; GraphPad Software, San Diego, CA) was used for the graphical presentation of data. Data were represented as mean ± standard error, and *p*-values less than 0.05 were considered statistically significant.

## 3. Results

### 3.1. Semen parameters

Before the commencement of the experiment, the founder dog was evaluated for the basic semen parameters. The volume of semen, the concentration of spermatozoa, %motility, and %progressive motility were 2.57 ± 0.23 mL, 1.73 ± 0.37 × 10^8^/mL, 77.33 ± 3.93%, and 62.67 ± 1.86%, respectively, for the founder dog. The values are the mean ± SE of three replications.

**Figure 1:**
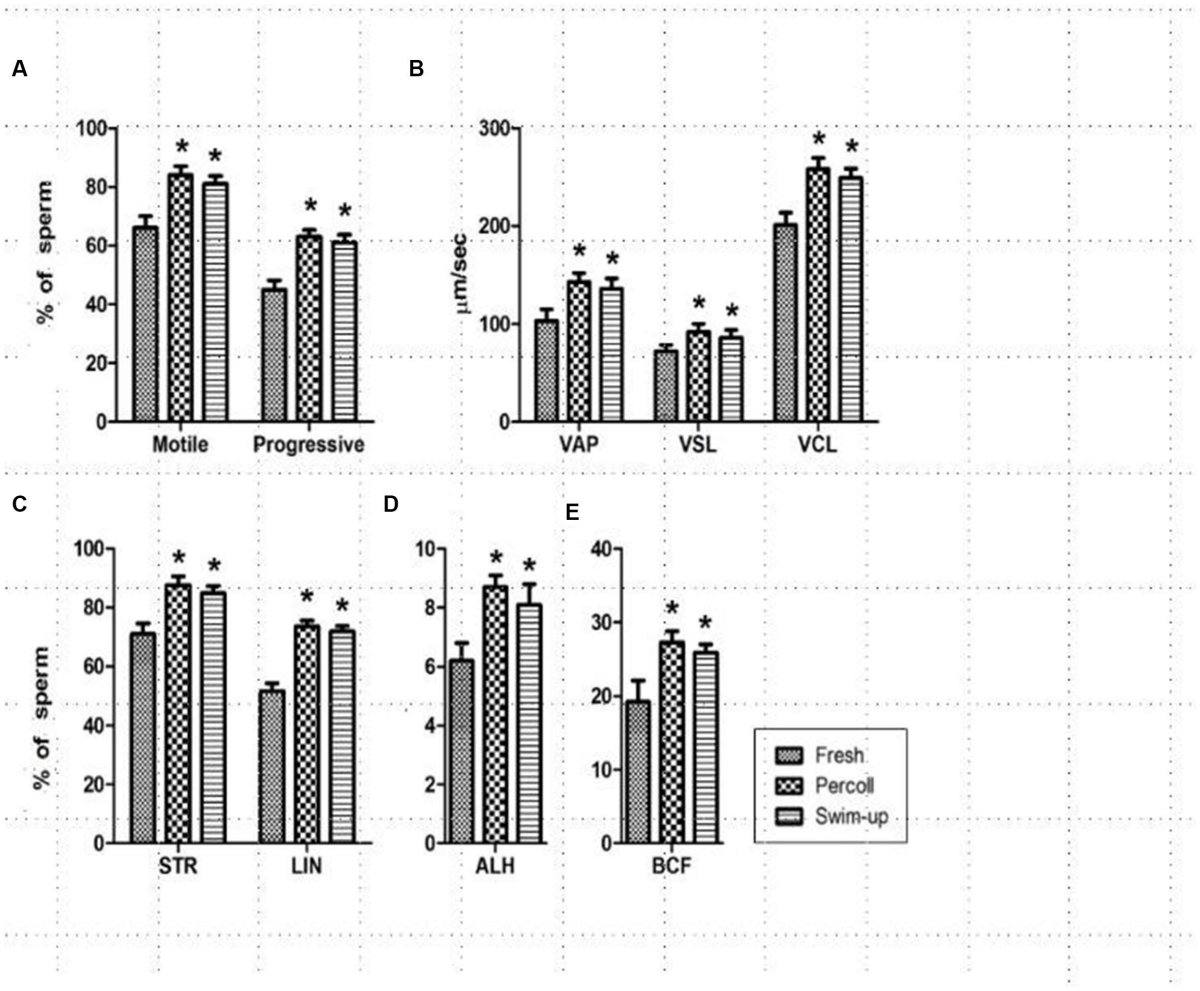
Changes in sperm track velocities after treatment. **(A)** Sperm motility and progressiveness; **(B)** average path velocity (VAP), straight-line velocity (VSL), and curvilinear velocity (VCL); **(C)** straightness (STR), linear coefficient (LIN); **(D)** amplitude of the lateral head displacement (ALH); **(E)** beat cross-frequency (BCF). ^*^ in the same column indicates a significant difference (*p* < 0.05). This experiment was repeated three times.

During the experiment, nine parameters, namely, motility, progressive motility, VAP, VSL, VCL, STR, LIN, mALH, and BCF were measured in the freshly diluted, Percoll gradient, and swim-up treatment groups and are presented in Figure 1. All parameters were significantly higher for the spermatozoa in the Percoll gradient and swim-up groups than those in the freshly diluted semen groups (*p* < 0.001).

### 3.2. Developmental competence of the IVF-derived embryos

To determine the pre-implantation developmental competence of the embryos generated by IVF, presumptive zygotes at the one-cell stage were transferred to surrogates with an estrous cycle similar to that of the egg donor. Embryos were recovered after 7 days, and their stage was categorized as cleaved (two-cell stage), morula, and blastocyst stage (Table 2). There were no significant differences in embryo recovery and development up to the morula stage among the different groups.

**Table 2:** Pre-implantation developmental competence of IVF-derived canine embryos *in vivo*.

Sperm treatment	No. of surrogates	Number of embryos				Number of embryos developed to blastocyst (%)^c^
		Transferred	Recovered (%)^b^	Cleaved (%)^c^	Morula (%)^c^	
Fresh	5	59 (11.8 ± 2.39)	44 (74.58)	18 (40.90)	11 (25.0)	0 (0.0)
Swim-up	5	65 (13.0 ± 1.87)	49 (75.38)	31 (63.27)	6 (12.25)	7 (14.29)^a^
Percoll gradient	5	70 (14.0 ± 1.87)	55 (78.57)	36 (65.46)	3 (5.46)	15 (27.27)^a^

a Values in different rows but in the same column marked with a superscript are significantly different (*p* < 0.05).

b The percentage of embryos recovered was calculated based on the number of embryos transferred.

c The percentage of embryos developed in each stage was calculated relative to the number of recovered embryos.

**Figure 2:**
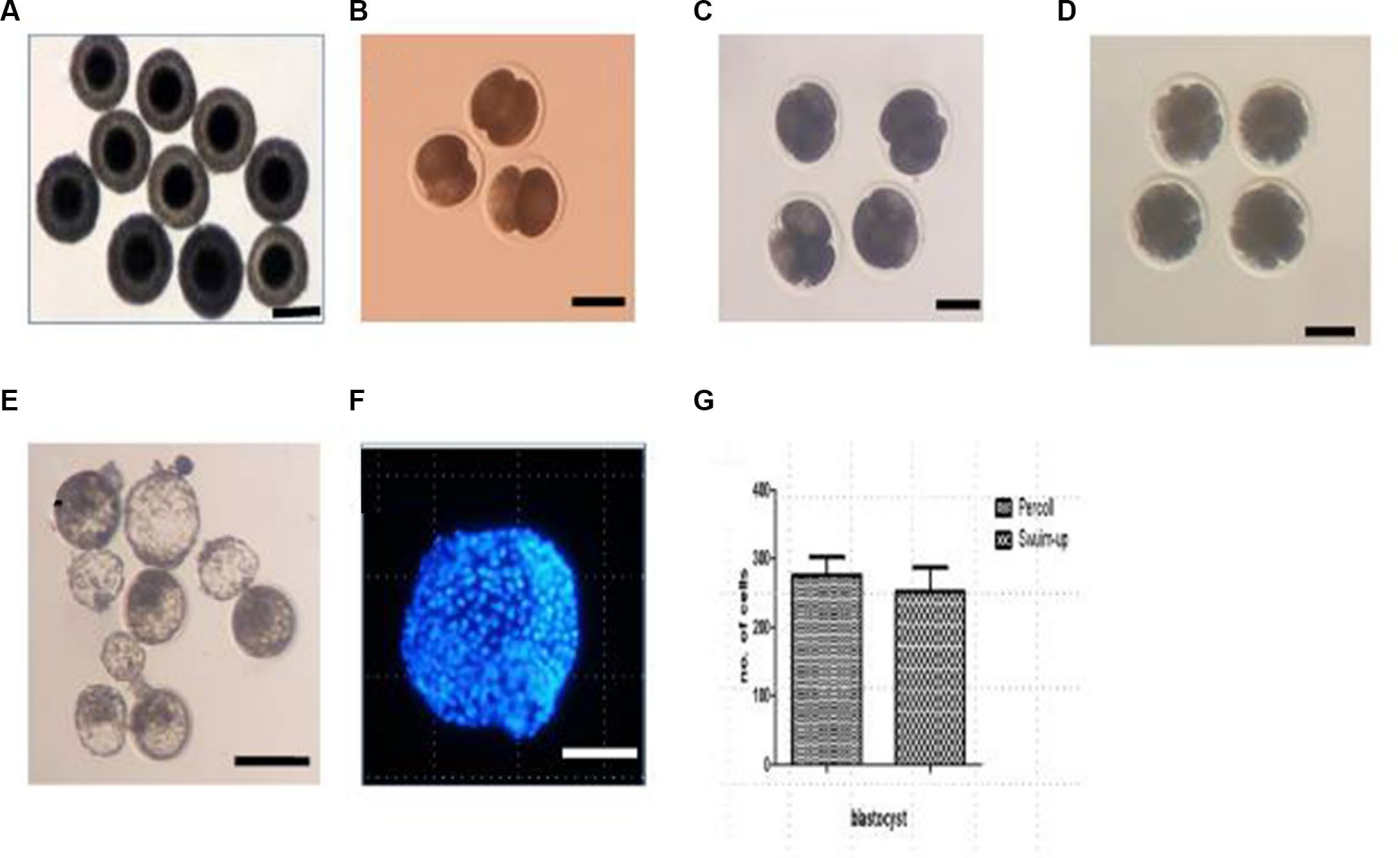
*In vivo* collection of oocytes and embryos at different developmental stages. **(A)** *In vivo* collected oocytes; **(B)** two-cell embryos; **(C)** four-cell embryos; **(D)** morula stage embryos; **(E)** blastocyst under bright field; **(F)** blastocyst under fluorescence light; **(G)** cell number in the blastocyst. Scale bar = 100 μM.

The blastocyst formation rate was significantly higher in the Percoll gradient (27.27%) and swim-up (14.29%) groups than in the freshly diluted group (0%; *p* < 0.05). Representative images of embryos and blastocysts derived by the IVF are shown in Figure 2. To determine the quality of the blastocysts, they were stained with Hoechst 33258, and the total cell numbers were counted. The average cell numbers in the blastocysts were 259.75 ± 26.24 and 237.41 ± 31.74 for the Percoll and swim-up, respectively (2G). However, the difference between these groups was not statistically significant.

### 3.3. Post-implantation developmental competence

The basic parameters for producing transgenic puppies are summarized in Table 3. We have retrieved 86 oocytes (12.29 ± 1.10/ dog) from 7 dogs; 65 presumptive zygotes were transferred to 5 dogs (13.4 ± 1.03 zygotes/ recipients) in the Percoll group and 1 recipient (20.0%) became pregnant. It had four (6.15%) sacs and delivered four (6.15%) live puppies by natural birth. Among the four puppies, two were female and two were male (Table 4). The birth weight was 575 g (AF, Alzheimer’s female AF1), 460 g (AF2), 550 g (AM, Alzheimer’s male AM3), and 450 g (AM4), respectively. The first parturition was initiated late on the 59th day post-embryo transfer and continued until early morning on the 60th day, leading to a discrepancy in the full-term gestation period between the first parturition and the others. No pregnancy was established in the swim-up group.

**Table 3:** Production rates of transgenic F1 puppies.

Parameter	Swim-up	Percoll gradient
Number of oocyte donors	7	7
Number of retrieved oocytes (mean±SE)	91 (13.0±1.12)	86 (12.29±1.10)
Number of surrogates	5	5
Total number of transferred embryos	69	65
Average number of presumptive zygotes per surrogate	13.8±0.86	13.4±1.03
Number of pregnant recipients (%)^a^	0	1 (20.0)
Number of sacs observed	0	4 (6.15)
Number of puppies born^b^	0	4 (6.15)

Values in different columns are not significant.

^a^Pregnancy rate was calculated relative to the number of surrogates.

^b^Offspring born was calculated relative to the number of embryos transferred.

**Table 4:** Pregnancy parameter and GFP expression of IVF offspring.

Cloned offspring	Gender	Gestation term (days)	Birth weight (g)	Age at death (days)	GFP expression
AF1	Female	59	575	N/A	(+)
AF2	Female	60	460	9	(+)
AM3	Male	60	550	N/A	(−)
AM4	Male	60	450	N/A	(−)

### 3.4. Germline transmission and parentage analysis

**Figure 3:**
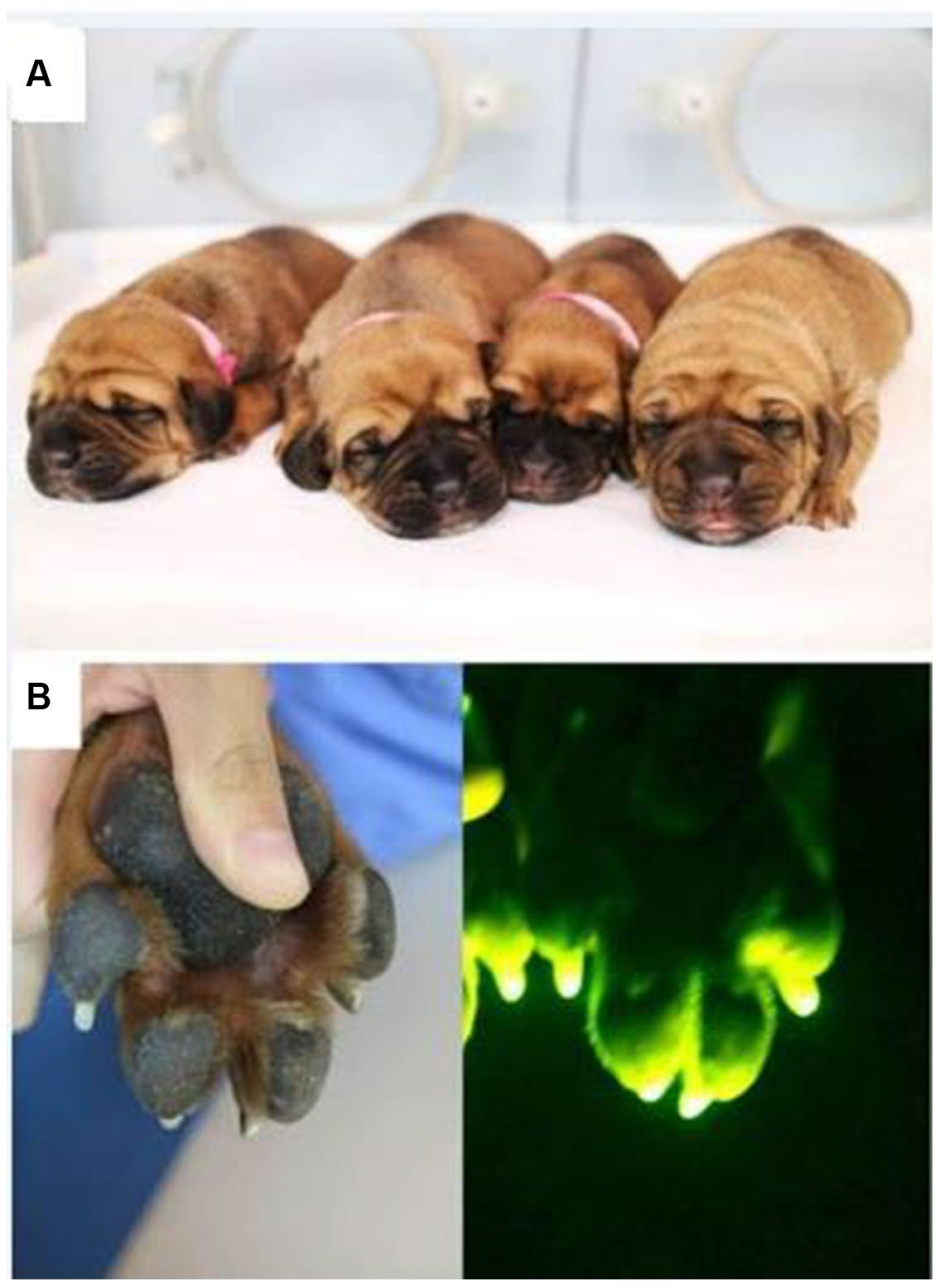
Photographs of **(A)** the transgenic IVF puppies and **(B)** GFP expression in their toes.

**Figure 4:**
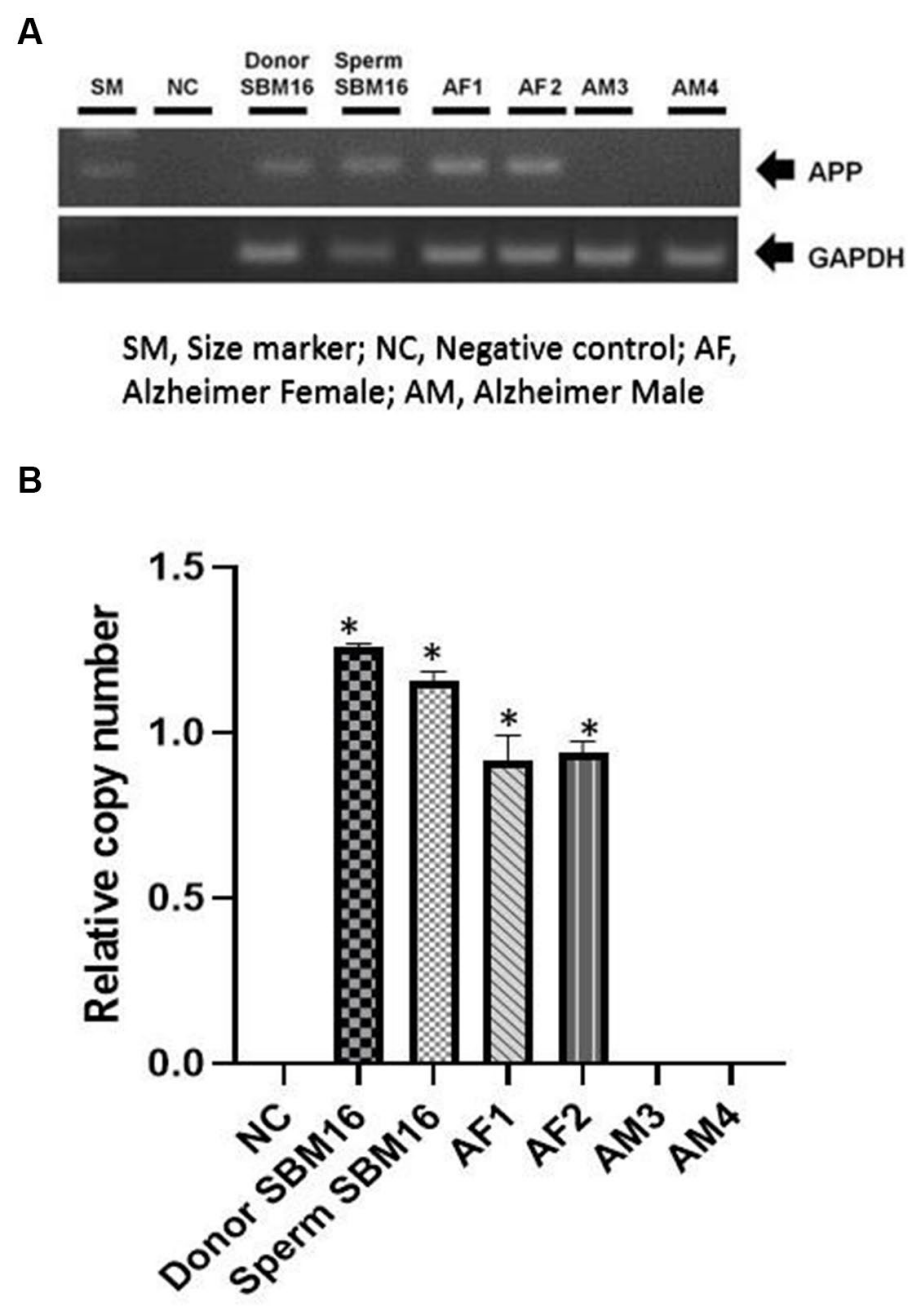
Confirmation of transgene expression in transgenic pups. Genomic DNA was obtained from puppies’ umbilical cords or age-matched non-transgenic puppies. **(A)** Chromosomal insertion of the mhAPP gene was confirmed using PCR. **(B)** mhAPP mRNA expression levels were measured using real-time PCR. SM, size marker; NC, negative control; *p < 0.05, **p < 0.01; relative gene expression levels are presented as a graph (mean  ±  SEM from triplicate evaluations in each sample).

Among the four puppies, two (50.0%) were found to transmit the transgene as observed by GFP fluorescence on the nail and toe (Figure 3). The integration and expression of the mhAPP transgene in the puppies were examined in the umbilical cords of all the IVF-derived puppies. The presence of the transgene (Figure 4A) and their expression (Figure 4B) were only observed in the GFP-positive puppies (AF1 and AF2 for the mhAPP gene). Microsatellite analysis of 18 canine loci revealed that the IVF-derived offspring were identical to their sperm and oocyte donors (raw data can be obtained by requesting the corresponding author).

## 4. Discussion

Our ultimate objective is to standardize a practical approach to generating transgenic puppies from transgenic founder dogs by germline transmission for biomedical research. SCNT offers a unique opportunity for the production of genetically modified animals. The first transgenic dog expressing red fluorescent protein was produced by the SCNT technique (15). We have reported the production of two transgenic puppies expressing the human phosphoenolpyruvate carboxykinase (hPEPCK) and the mhAPP gene using the SCNT procedure (11, 26). Herein, we report the production of mhAPP transgenic puppies using germline transmission using IVF.

SCNT is a widely used and reliable technique to produce transgenic animals. However, it is a highly technical, time-consuming, and costly procedure. Deliberate efforts were made to overcome these issues related to SCNT. The most favorable solution to produce many transgenic animals in a shorter time could be producing a founder animal by SCNT and propagating that founder animal by germline transmission either by natural mating or by assisted reproductive techniques such as artificial insemination and IVF. Germline transmission could be a great help in the mass production of transgenic animals for biomedical research as it simplifies the production process and significantly reduces the time and cost of cloning.

The reproductive performances of both cloned and transgenic animals are reported to be similar to aged-matched non-cloned animals. Park et al. (27) evaluated the reproductive activity of male and female cloned dogs. They reported that male cloned dogs had sperm motility parameters within the reference range, and cloned female dogs had standard patterns of reproductive hormones. Furthermore, viable puppies were obtained when cloned females were artificially inseminated using semen collected from a cloned dog.

The motility of sperm, especially progressive motility, is a commonly used parameter for semen quality. In addition to motility, another vigor parameter, such as velocity (average path, curvilinear, or straight), is also essential in predicting the fertilizing capacity of sperm. In this study, we used CASA to assess the quality of semen specimens prepared using three different methods. Rijsselaere et al. (24) reported that with proper internal image settings, this analyzer could objectively assess canine semen quality for research and practical purposes. CASA can significantly improve the accuracy and precision of dog semen analysis methods. Furthermore, CASA has been successfully used in other mammalian species, such as bulls, boars, and humans, to improve IVF outcomes (28, 29).

Several reports describing mammalian sperm preparation using swim-up and Percoll gradient methods have been published (28, 29). However, these reports are inconsistent. Percoll separation has been reported to be more effective for selecting highly motile sperm in cattle (23), but buffalo and stallion sperm were found of higher quality when prepared using the swim-up method (30, 31). A direct comparison of the efficiency of motile spermatozoa separation from the same sample has not been conducted in dogs. Our study revealed that both the swim-up and Percoll methods separated the highest number of motile and progressively motile sperm, compared with freshly diluent semen.

In this study, freshly diluted semen failed to produce any blastocysts, while the swim-up method produced 14.29% blastocysts. However, no pregnancy was established. Freshly diluted semen may have much more debris and abnormal or dead spermatozoa which are reflected in less motility, progressive motility, and other parameters. Ultimately, no blastocyst was produced from freshly diluted semen. The Percoll method produced 27.27% blastocyst; successful pregnancy and live puppies were born in this method. Only the Percoll method was shown to produce live offspring in this study. Motility and progressive motility were better for the Percoll-prepared spermatozoa than the swim-up procedure. Higher blastocyst rates in the Percoll protocol may result in successful pregnancies. It is worth mentioning that the pregnancy rate following artificial insemination is significantly influenced by the quality of the semen, especially with regard to the motility and progressiveness of sperm (32). Ren et al. (33) compared four different methods of semen preparation and reported the highest pregnancy rates following intrauterine insemination using sperm prepared via the Percoll method. They argued that the Percoll method produced sperm with superior VAP and VSL, which might explain the higher pregnancy rates.

The availability of mature oocytes is the main limiting factor for biotechnological research in canines. *In vivo*, matured oocytes are considered the primary source of oocytes as a reliable *in vitro* system for canines is yet to be developed (13, 14). Mammals usually ovulate mature oocytes, while dogs ovulate immature oocytes at their germinal vesicle stage of development (34). As a result, oocytes need 3 to 5 days in the fallopian tube to reach the metaphase II stage, which is considered mature oocytes (35). Therefore, mature canine oocytes are collected by flushing the fallopian tubes (20). In the present experiment, we also collected *in vivo* matured oocytes from naturally cyclic bitches by flushing the fallopian tubes, and we retrieved, on average, 12.29 oocytes per dog. Nagashima et al. (17) collected *in vivo* matured oocytes by flushing the excised oviducts of 46 beagle dogs and reported an average of 4.77 oocytes per dog, which is lower than our retrieval rate. The differences in oocyte recovery may be due to breed variation. Nagashima et al. (17) used purebred beagle dog, which is medium-sized, while we used mixed-breed dogs, much larger than beagle dogs.

The first live puppy generated from IVF-derived embryos was reported by Nagashima et al. (17), where they emphasized using *in vivo* matured oocytes and suitable protocol for sperm preparation. We reported here that only the Percoll gradient method was able to produce transgenic puppies by germline transmission; 4 live puppies (6.15%) were born from 1 pregnant recipient out of 5 recipients. We observed 50.0% germline transmission to the F1 generation; among the 4 puppies, 2 were found to transmit GFP on their nail and toe. Furthermore, as revealed on RT-PCR, GFP-positive puppies showed genomic integration of mhAPP transgene. Puppies expressing the mhAPP gene could produce Alzheimer’s disease-like symptoms such as cognitive dysfunction. These transgenic dogs are useful for understanding the developmental and pathological phenomena of Alzheimer’s disease and hence helpful in the development of novel therapies. Germline transmission in cloned transgenic animals is reported in various species, such as bovine (36), porcine (37), caprine (38), and marmoset (39). Hong et al. (15) reported that 52.6% of F1 and F2 offspring showed emission of red fluorescence protein in their skin and claws, which were readily apparent even under bright-field illumination.

The birth weight of our transgenic puppies was from 450 g to 575 g. They were healthy, and no congenital anomalies were detected. However, in the course of the experiment, one puppy (AM2) died at the age of 9 days. Post-mortem findings revealed the cause of death as non-specific. It is unlikely that the death of this puppy was due to the detrimental effect of the transgene because the other siblings carrying the same transgene are still alive and healthy.

## 5. Conclusion

Successful germline transmission of a transgene in canines could be obtained using the IVF technique. Semen prepared by the Percoll method can produce live transgenic puppies by germline transmission as two out of four offspring showed integration of the transgene into their genome. Our result will benefit the propagation of transgenic dogs easily, economically, and efficiently, which will foster disease model studies in humans.

## Data availability statement

The original contributions presented in the study are included in the article; further inquiries can be directed to the corresponding author.

## Ethics statement

The animal studies were approved by Abu Dhabi Biotech Research Foundation, Republic of Korea. The studies were conducted in accordance with the local legislation and institutional requirements. Written informed consent was obtained from the owners for the participation of their animals in this study.

## Author contributions

MH, Y-BS, YWJ, YIJ, MNK, EJC, KBP, YRB and YB designed and conducted the experiment. MH and DK wrote and revised the manuscript. Y-BS and YWJ analyzed the data. WH designed and supervised the whole experiment, and revised the manuscript. All authors contributed to the article and approved the submitted version.

## References

[1] Tang L González R Dobrinski I. Germline modification of domestic animals. Anim Reprod. (2015) 12:93–104. PMID: 27390591 PMC4933526

[2] Lampreht TU Horvat S Cemazar M. Transgenic mouse models in cancer research. Front Oncol. (2018) 8:268. doi: 10.3389/fonc.2018.00268 30079312 PMC6062593

[3] Tsai KL Clark LA Murphy KE. Understanding hereditary diseases using the dog and human as companion model systems. Mamm Genome. (2007) 18:444–51. doi: 10.1007/s00335-007-9037-1, PMID: 17653794 PMC1998873

[4] Sargan DR. IDID: inherited diseases in dogs: web-based information for canine inherited disease genetics. Mamm Genome. (2004) 15:503–6. doi: 10.1007/s00335-004-3047-z, PMID: 15181542

[5] Davis BW Ostrander EA. Domestic dogs and cancer research: a breed-based genomics approach. ILAR J. (2014) 55:59–68. doi: 10.1093/ilar/ilu017, PMID: 24936030 PMC4158346

[6] Gordon J Scangos G Plotkin D Barbosa J Ruddle F. Genetic transformation of mouse embryos by microinjection of purified DNA. Proc Natl Acad Sci U S A. (1980) 77:7380–4. doi: 10.1073/pnas.77.12.7380, PMID: 6261253 PMC350507

[7] Pereyra-Bonnet F Gibbons A Cueto M Sipowicz P Fernandez-Martin R Salamone D. Efficiency of sperm-mediated gene transfer in the ovine by laparoscopic insemination, in vitro fertilization and ICSI. J Reprod Dev. (2011) 57:188–96. doi: 10.1262/jrd.10-063A, PMID: 21079375

[8] Lois C Hong EJ Pease S Brown EJ Baltimore D. Germline transmission and tissue-specific expression of transgenes delivered by lentiviral vectors. Science. (2002) 295:868–72. doi: 10.1126/science.1067081, PMID: 11786607

[9] Doudna JA Charpentier E. Genome editing. The new frontier of genome engineering with CRISPR-Cas9. Science. (2014) 346:1258096. doi: 10.1126/science.1258096 25430774

[10] Polejaeva IA Campbell KH. New advances in somatic cell nuclear transfer: application in transgenesis. Theriogenology. (2000) 53:117–26. doi: 10.1016/S0093-691X(99)00245-9, PMID: 10735067

[11] Lee GS Jeong YW Kim JJ Park SW Ko KH Kang M. A canine model of Alzheimer's disease generated by overexpressing a mutated human amyloid precursor protein. Int J Mol Med. (2014) 33:1003–12. doi: 10.3892/ijmm.2014.1636, PMID: 24481173

[12] Rocchi A Pellegrini S Siciliano G Murri L. Causative and susceptibility genes for Alzheimer's disease: a review. Brain Res Bull. (2003) 61:1–24. doi: 10.1016/S0361-9230(03)00067-4, PMID: 12788204

[13] Luvoni GC. Current Progress on assisted reproduction in dogs and cats: in vitro embryo production. Reprod Nutr Dev. (2000) 40:505–12. doi: 10.1051/rnd:2000114, PMID: 11140820

[14] Van Soom A Rijsselaere T Filliers M. Cats and dogs: two neglected species in this era of embryo production in vitro? Reprod Domest Anim. (2014) 49:87–91. doi: 10.1111/rda.12303, PMID: 24947866

[15] Hong SG Kim MK Jang G Oh HJ Park JE Kang JT. Generation of red fluorescent protein transgenic dogs. Genesis. (2009) 47:314–22. doi: 10.1002/dvg.20504, PMID: 19358155

[16] Ng FL Liu DY Baker HW. Comparison of Percoll, mini-Percoll and swim-up methods for sperm preparation from abnormal semen samples. Hum Reprod. (1992) 7:261–6. doi: 10.1093/oxfordjournals.humrep.a137628, PMID: 1315792

[17] Nagashima JB Sylvester SR Nelson JL Cheong SH Mukai C Lambo C. Live births from domestic dog (*Canis familiaris*) embryos produced by in vitro fertilization. PLoS One. (2015) 10:e0143930. doi: 10.1371/journal.pone.0143930, PMID: 26650234 PMC4674105

[18] Bollendorf A Check JH Katsoff D Lurie D. Comparison of direct swim-up, mini-Percoll, and Sephadex G10 separation procedures. Arch Androl. (1994) 32:157–62. doi: 10.3109/01485019408987781 8166579

[19] Van der Zwalmen P Bertin-Segal G Geerts L Debauche C Schoysman R. Sperm morphology and IVF pregnancy rate: comparison between Percoll gradient centrifugation and swim-up procedures. Hum Reprod. (1991) 6:581–8. doi: 10.1093/oxfordjournals.humrep.a137383, PMID: 1918311

[20] Hossein MS Jeong YW Kim S Kim JJ Park SW Jeong CS. Protocol for the recovery of in vivo matured canine oocytes based on once daily measurement of serum progesterone. Cloning Stem Cells. (2008) 10:403–8. doi: 10.1089/clo.2008.0001, PMID: 18578591

[21] Lee BC Kim MK Jang G Oh HJ Yuda F Kim HJ. Dogs cloned from adult somatic cells. Nature. (2005) 436:641. doi: 10.1038/436641a, PMID: 16079832

[22] Mahi CA Yanagimachi R. Capacitation, acrosome reaction, and egg penetration by canine spermatozoa in a simple defined medium. Gamete Res. (1978) 1:101–9. doi: 10.1002/mrd.1120010203

[23] Parrish JJ Krogenaes A Susko-Parrish JL. Effect of bovine sperm separation by either swim-up or Percoll method on success of in vitro fertilization and early embryonic development. Theriogenology. (1995) 44:859–69. doi: 10.1016/0093-691x(95)00271-9 16727781

[24] Rijsselaere T Van Soom A Maes D de Kruif A. Effect of technical settings on canine semen motility parameters measured by the Hamilton-Thorne analyzer. Theriogenology. (2003) 60:1553–68. doi: 10.1016/S0093-691X(03)00171-7, PMID: 14519475

[25] Jeong YH Lu H Park CH Li M Luo H Kim JJ. Stochastic anomaly of methylome but persistent SRY hypermethylation in disorder of sex development in canine somatic cell nuclear transfer. Sci Rep. (2016) 6:31088. doi: 10.1038/srep31088 27501986 PMC4977463

[26] Jeong YW Lee GS Kim JJ Park SW Ko KH Kang M. Establishment of a canine model of human type 2 diabetes mellitus by overexpressing phosphoenolpyruvate carboxykinase. Int J Mol Med. (2012) 30:321–9. doi: 10.3892/ijmm.2012.993, PMID: 22580743

[27] Park JE Hong SG Kang JT Oh HJ Kim MK Kim MJ. Birth of viable puppies derived from breeding cloned female dogs with a cloned male. Theriogenology. (2009) 72:721–30. doi: 10.1016/j.theriogenology.2009.05.007, PMID: 19580995

[28] Cancel AM Lobdell D Mendola P Perreault SD. Objective evaluation of hyperactivated motility in rat spermatozoa using computer-assisted sperm analysis. Hum Reprod. (2000) 15:1322–8. doi: 10.1093/humrep/15.6.1322, PMID: 10831563

[29] Farrell PB Foote RH McArdle MM Trouern-Trend VL Tardif AL. Media and dilution procedures tested to minimize handling effects on human, rabbit, and bull sperm for computer-assisted sperm analysis (CASA). J Androl. (1996) 17:293–300. doi: 10.1002/j.1939-4640.1996.tb01785.x PMID: 8792220

[30] Mehmood A Anwar M Naqvi SM. Motility, acrosome integrity, membrane integrity and oocyte cleavage rate of sperm separated by swim-up or Percoll gradient method from frozen-thawed buffalo semen. Anim Reprod Sci. (2009) 111:141–8. doi: 10.1016/j.anireprosci.2008.02.011, PMID: 18378413

[31] Sieme H Martinsson G Rauterberg H Walter K Aurich C Petzoldt R. Application of techniques for sperm selection in fresh and frozen-thawed stallion semen. Reprod Domest Anim. (2003) 38:134–40. doi: 10.1046/j.1439-0531.2003.00416.x, PMID: 12654024

[32] Mickelsen WD Memon MA Anderson PB Freeman DA. The relationship of semen quality to pregnancy rate and litter size following artificial insemination in the bitch. Theriogenology. (1993) 39:553–60. doi: 10.1016/0093-691X(93)90397-N, PMID: 16727234

[33] Ren SS Sun GH Ku CH Chen DC Wu GJ. Comparison of four methods for sperm preparation for IUI. Arch Androl. (2004) 50:139–43. doi: 10.1080/01485010490425566 15204678

[34] Reynaud K Fontbonne A Saint-Dizier M Thoumire S Marnier C Tahir MZ. Folliculogenesis, ovulation and endocrine control of oocytes and embryos in the dog. Reprod Domest Anim. (2012) 47:66–9. doi: 10.1111/rda.12055, PMID: 23279468

[35] Luvoni GC Chigioni S Allievi E Macis D. Factors involved in vivo and in vitro maturation of canine oocytes. Theriogenology. (2005) 63:41–59. doi: 10.1016/j.theriogenology.2004.03.004, PMID: 15589272

[36] Brophy B Smolenski G Wheeler T Wells D L’Huillier P Laible G. Cloned transgenic cattle produce milk with higher levels of beta-casein and kappa casein. Nat Biotechnol. (2003) 21:157–62. doi: 10.1038/nbt783, PMID: 12548290

[37] Brunetti D Perota A Lagutina I Colleoni S Duchi R Calabrese F. Transgene expression of green fluorescent protein and germ line transmission in cloned pigs derived from in vitro transfected adult fibroblasts. Cloning Stem Cells. (2008) 10:409–19. doi: 10.1089/clo.2008.0036, PMID: 18823265

[38] Keefer CL Baldassarre H Keyston R Wang B Bhatia B Bilodeau AS. Generation of dwarf goat (*Capra hircus*) clones following nuclear transfer with transfected and nontransfected fetal fibroblasts and in vitro-matured oocytes. Biol Reprod. (2001) 64:849–56. doi: 10.1095/biolreprod64.3.849, PMID: 11207200

[39] Sasaki E Suemizu H Shimada A Hanazawa K Oiwa R Kamioka M. Generation of transgenic non-human primates with germline transmission. Nature. (2009) 459:523–7. doi: 10.1038/nature08090, PMID: 19478777

