# Supplementary material for: Production of transgenic first filial puppies expressing mutated human amyloid precursor protein gene
Source: Front Vet Sci. 2023 Oct 30;10:1227202. doi: 10.3389/fvets.2023.1227202 (PMC10642565; doi:10.3389/fvets.2023.1227202)
Supplement: Supplementary file 1 [file Table_1.DOCX]

**Supplementary Table 1. Component and concentration of washing-TALP, Cap-TALP and IVF-TALP**

| **Component** | **Washing-TALP** | **Cap-TALP** | **IVF-TALP** |
| --- | --- | --- | --- |
| **Salt:**  NaCl (mg/ml)  KCl (mg/ml) | 4.88  0.36 | 4.88  0.36 | 4.88  0.36 |
| **Buffer:**  Hepes (mg/ml)  KH_2_PO_4_ (mg/ml)  NaHCO_3_ (mg/ml) | 4.77  0.162 | 14.30  0.162  3.159 | 4.77  0.162  3.159 |
| **Energy Substrate:**  Na Pyruvate (mg/ml)  Na Lactate 60% Syrup (ul/ml)  Glucose (mg/ml)  Bovine Serum Albumin (mg/ml)  NEAA (ul/ml) | 0.028  3.38  0.50 | 0.028  3.38  0.50  2.00 | 0.028  3.38  0.50  2.00  1.95 |
| **Sperm Capacitation:**  CaCl_2_ 2H_2_O (mg/ml)  MgCl_2_6H_2_O (mg/ml)  Hypotaurine (mg/ml) |  | 0.189  0.099  0.099 | 0.189  0.099  0.099 |
| **Antibiotics:**  Streptomycin Sulphate (mg/ml)  Kenmamycin (mg/ml) | 0.05 | 0.07 | 0.07 |
| PH | 7.4 | 7.4 | 7.6 |
| Osmolarity mOsm | 280 | 280 | 280 |
